# Genetic analysis of the Hungarian draft horse population using partial mitochondrial DNA D-loop sequencing

**DOI:** 10.7717/peerj.4198

**Published:** 2018-01-31

**Authors:** Nikolett Csizmár, Sándor Mihók, András Jávor, Szilvia Kusza

**Affiliations:** Institute of Animal Husbandry, Biotechnology and Nature Conservation, University of Debrecen, Debrecen, Hungary

**Keywords:** *Equus caballus*, Genetic diversity, mtDNA, D-loop region, Hungarian draft horse

## Abstract

**Background:**

The Hungarian draft is a horse breed with a recent mixed ancestry created in the 1920s by crossing local mares with draught horses imported from France and Belgium. The interest in its conservation and characterization has increased over the last few years. The aim of this work is to contribute to the characterization of the endangered Hungarian heavy draft horse populations in order to obtain useful information to implement conservation strategies for these genetic stocks.

**Methods:**

To genetically characterize the breed and to set up the basis for a conservation program, in the present study a hypervariable region of the mitochrondial DNA (D-loop) was used to assess genetic diversity in Hungarian draft horses. Two hundred and eighty five sequences obtained in our laboratory and 419 downloaded sequences available from Genbank were analyzed.

**Results:**

One hundred and sixty-four haplotypes and thirty-six polymorphic sites were observed. High haplotype and nucleotide diversity values (*H*_*d*_ = 0.954 ± 0.004; *π* = 0.028 ± 0.0004) were identified in Hungarian population, although they were higher within than among the different populations (*H*_*d*_ = 0.972 ± 0.002; *π* = 0.03097 ± 0.002). Fourteen of the previously observed seventeen haplogroups were detected.

**Discussion:**

Our samples showed a large intra- and interbreed variation. There was no clear clustering on the median joining network figure. The overall information collected in this work led us to consider that the genetic scenario observed for Hungarian draft breed is more likely the result of contributions from ‘ancestrally’ different genetic backgrounds. This study could contribute to the development of a breeding plan for Hungarian draft horses and help to formulate a genetic conservation plan, avoiding inbreeding while.

## Introduction

In recent decades, various types of animal species have been investigated with special emphasis on improving the efficiency of selection programs and the use of modern molecular genetic methods has increased considerably. As a consequence of the development of feeding technologies and the acceleration of transport and communication, local native breeds have been worldwide replaced by modern, high-productivity varieties. However, the genetic value of gene conservation divided the fate of varieties: those having excellent secondary traits and rare alleles resulting in considerable diversity contribute to both the current and future preservation of desirable properties (Notter, 1999; Bruford, Bradley & Luikart, 2003; Toro, Fernandez & Caballero, 2009; Bruford et al., 2015). It is generally accepted that detailed molecular genetic data describing inter- and intraspecies diversity are essential for the effective management of genetic resources among economic animal varieties (Weitzman, 1993; Hall & Bradley, 1995; Barker, 1999; Ruane, 2000; Bruford, Bradley & Luikart, 2003; Simianer, 2005; Toro & Caballero, 2005; Toro, Fernandez & Caballero, 2009; Ginja et al., 2013). These data as well as the continuous development of technology offer many new opportunities for researchers. In molecular genetic studies serving gene conservation, breeds are the basic units (Groeneveld et al., 2010).

In phylogenetic studies of mammalian species/groups, mitochondrial DNA is a widely used molecular marker. The entire horse (*Equus caballus*) mitochondrial genome sequence has been available since 1994 (Xu & Arnason, 1994). The species is represented worldwide by more than 58 million animals (FAOSTAT, 2010). Extant horses can be traced back to the domestication diversification process, which began at least 5,000–6,000 years ago in the Eurasian steppe region (Ludwig et al., 2009; Outram et al., 2009; Cieslak et al., 2010; Lippold et al., 2011; Schubert et al., 2014; Librado & Rozas, 2016). A significant portion of the observed diversity of modern maternal lines was also observed at the time of domestication and throughout time; there is an extensive sharing of maternal lineages among horse populations worldwide, while high mtDNA diversity is of ancient origin (Keyser-Tracqui et al., 2005). Due to these evolutionary processes, modern horses today form close populations, whose individuals carry unique bloodlines and/or phenotypes (Petersen et al., 2013). After the Second World War various horse populations declined Europe-wide, leading to reduction of genetic diversity. However, in recent years the issue of the preservation of genetic diversity has gained special emphasis on the international level, and one of the main considerations in this area of scientific research activities is to preserve the biodiversity of local breeds (Georgescu & Costache, 2012; Sziszkosz et al., 2016). The import of heavy horses to Hungary started in the second half of the 19th century. These were mainly stallions of the Belgian, Percheron, Breton and Ardens breeds. Until after World War II, no organized breeding of heavy horses existed in Hungary. After World War II, there was a great need for horses to be used in field work on farms and also in transportation in Hungary. The foundation stock of this breed initially were native Hungarian mares which were bred with various other breeds such as Noriker, Percheron and Ardennes, and also with the available native Hungarian stallions. As a result of breeding work, a few local types (Muraközi and Pinkafői) were developed. The Hungarian Draft Horse Breeders National Association has records of approximately 800 mares today. The maternal side anestry of some individuals of the current stock contains unknowns in 3rd–4th generations, since brand-marking and pedigree registration was enforced only in 1993. In the maternal side of the Hungarian cold-blooded horse breeding stock, original pedigree documentation is missing and the founding stallions of the breed are unknown. Therefore, it is especially important to explore the genetic background of the remaining stock and to get a first insight into the Hungarian population genetics.

## Materials and Methods

### Ethics statement

DNA sampling was limited to the collection of hairs pulled from the mane or tail by the horse owner or researcher. All animal work was conducted in accordance with the international and Hungarian national governing bodies (The Hungarian Animals Breeders Association—HABA, and Department of Operative Techniques and Surgical Research in Debrecen). All horses in this study were client-owned, no harmful invasive procedure was performed on them, there was no animal experimentation according to the legal definitions in Europe (Subject 5f of Article 1, Chapter I of the Directive 2010/63/UE of the European Parliament and of the Council), and in Hungary (40/2013. (II. 14.) Government Decree on animal research and therefore no ethical committee approval was required.

### Samples

Two hundred eighty five samples from registered mares from all over Hungary representing 35.63% of the Hungarian draft horse population were used. Hair samples from the tail complete with follicles were used and were stored airtight until examinations at the laboratory at room temperature. Following verbal consent, each breeder contributed to the tests as well as for the study being prepared. Samples were examined in the Laboratory of Animal Genetics at the University of Debrecen. Genomic DNA was extracted from hair-root samples (FAO/IAEA, 2004), and was carried out following a Chelex-based protocol (Walsh, Metzger & Higuchi, 2013).

### Genbank sequences

We downloaded 419 sequences from 52 different breeds available at Genbank. We considered the presumed founding ancestors of Hungarian draft (KY512807–KY513091), 83 sequences examined by Achilli et al. (2012) and also used Rhineland Heavy draft, Noriker, Turkoman Akhal Teke, Italian heavy draught, Breton, Arabian, Finn horse, Polish primitive, Hucul, Zemaitukai heavy type, Shire, Vladimir draught horse, Clydesdale, Iranian, Trakehner, Caspian Pony, Polish heavy, Maremmano, Posavina, Akhal teke, Shetland pony, Pura Raza Espanola, Croatian heavy draft, Murinsulaner, Fell, Icelandic Horse, Norwegian Fjord, Romanian draft horse, Percheron, Lithuanian heavy drought, Oldenburg, Andalusian, Silesian, Italian, Scottish Highland, Belgian, Thoroughbred horse, American Paint horse, Hanovarian, Wielkopolski, Syrian, Giara horse, Holstein, Przewalskii, Gotland, Suffolk Punch, Westfalian, Chincoteague pony, Cleveland bay horse, Saddlebred and Exmoor pony populations with distant origins. Genbank Accession numbers for these sequences are the following: AB329597, AF064632–AF431969, AJ413825–AJ413900, AY246186–AY575139, DQ324048, EF014970–EF014989, EF494073–EF494083, EF495133–EF495151, EU093045–EU093073, EU256571–EU256622, GQ119632–GQ119636, GU339390, GU563634, GU563651, GU563669–GU563711, HQ439455–HQ593058, HQ848967–HQ848977, JN398377–JN398457, KC847166, KF192350–KF192499, KF849272–KF849290, KT757760–KT757761.

### mtDNA amplification, sequencing and analysis

Based on the published horse mtDNA sequence (Xu & Arnason, 1994), primers were designed for amplifying a 398-bp fragment containing the most variable segment of horse mtDNA, Forward 5′-CCCCCACATAACACCATACC-3′, Reverse 5′-AGACAGGCATCCCCCTAGAT-3′. PCR amplifications were performed in 30 µL reaction volumes. Amplification mixture was as follows: 5 µl isolated genomic DNA, 8.8 µl dNTP (25 mM)/Fermentas, 1 µl GoTaq Flexi Buffer Promega, 8.2 µl MgCl_2_ (25 mM) Promega, 1 µl forward and 1 µl reverse primer (10 pmol/ µl) Sigma, 5 µl dH_2_O. The reaction mixture was incubated at 95 °C for 10 min, followed by 35 cycles each consisting of 20 s denaturation at 95 °C, 30 s annealing at 62 °C, 30 s of extension at 70 °C and then a final 10min extension at 72 °C. Sequencing was done by the Macrogen Company (The Netherlands, Amsterdam). Sequences were assembled and truncated to a length of 222 bp (between positions 15,531 and 15,752) to maximize sample size comparisons. The correct reading of nucleotides and the comparison of sequences were done with CodonCodeAlignerV.6.0.2., whereas statistical analysis was performed with two versions of Mega (Mega6 (Tamura et al., 2013) and Mega7.0 (Kumar, Stecher & Tamura, 2016), with DnaSP5.1. (Librado & Rozas, 2009) and Network 5.0. (Bandelt, Forster & Rohl, 1999). The DnaSP5.1. software was used for calculating the total number of haplotypes, haplotype (*H* ± SD) and nucleotide (*π* ± SD) diversities. Genetic distances among different mtDNA haplotypes were calculated by the two-parameter method of Kimura (Kimura, 1980). Molecular Phylogenetic analysis was done by Maximum Likelihood method based on the Tamura-Nei model (Tamura & Nei, 1993). The initial trees for the heuristic search were obtained by applying the Neighbor-Joining method to a matrix of pairwise distances estimated using the Maximum Composite Likelihood (MCL) approach. A discrete Gamma distribution was used to model evolutionary rate differences among sites (5 categories (+G, parameter = 0.1234)). We used Arlequin 3.5.2.2. (Excoffier & Lischer, 2010) software for calculating pairwise *F*_ST_ values (and 5% significance levels) and detecting shared haplotypes among populations. Median joining networks were constructed using NETWORK version 5.0.0.1 (Bandelt, Forster & Rohl, 1999). Calculations were performed under the following conditions: deletions or insertions were double weighted, transition/transversion ratio = 6,5, default setting of Epsilon (0) was chosen.

## Results

### Indices of the genetic diversity of the Hungarian population

The haplotypes obtained were compared with an *Equus caballus* reference sequence available from Genbank (accession number X79547) and also used by Hill et al. (2002). Analysis of the mitochondrial control region sequence (part of the mitochondrial HVR I) from 285 individuals—(KY512807–KY513091) identified 55 haplotypes based on 35 variable nucleotide sites (222-bp sequence). Thirty-five polymorphic sites were detected, which represented 16.98% of the total mtDNA sequence analyzed (222 bp). The average ratios of the four nucleotides A, T, C, G were 32.7%, 28.1%, 27.4%, and 11.8%, respectively. We observed high haplotype and nucleotide diversity values (*H*_*d*_ = 0.954 ± 0.004; *π* = 0.034 ± 0.018). The average number of pairwise differences was *k* = 5.77497. During sequence analysis we considered only 204 from the included 222 positions, after excluding sites with gaps. Among the 55 haplotypes identified in these 204-bp sequences, we observed 22 unique haplotypes (i.e., found only in a single animal), whereas the most frequent haplotype was #34 identified in thirty individuals. Eight haplotypes comprise 55.78% of the total sequences, with the remaining 47 haplotypes including less than ten individuals in each group. The details of the variable positions in basepairs 15,531–15,752 are given in Table S1. Altogether 169 polymorphic sites were identified in this D-loop fragment across all horse populations (19 indels), representing a total of 164 different haplotypes. Thus the average percentage of polymorphic sites was 79.24% for all DNA sequences analyzed. High diversity values were observed among the total number of breeds (*H*_*d*_ = 0.972 ± 0.002; *π* = 0.03097 ± 0.002). The average number of pairwise differences was *k* = 6.164. Intra- and inter-breed variation is summarized in Table S2.

The lowest nucleotide diversities were found in Giara and Icelandic Horse (*π* = 0.0057 ± 0.008), whereas the highest values were in Fell horse (*π* = 0.047 ± 0.05). We observed the greatest number of polymorphic sites in Clydesdale, where the highest number of insertion/deletion positions (11) and transitions (42) as well as transversions (127) occurred.

**Figure 1:**
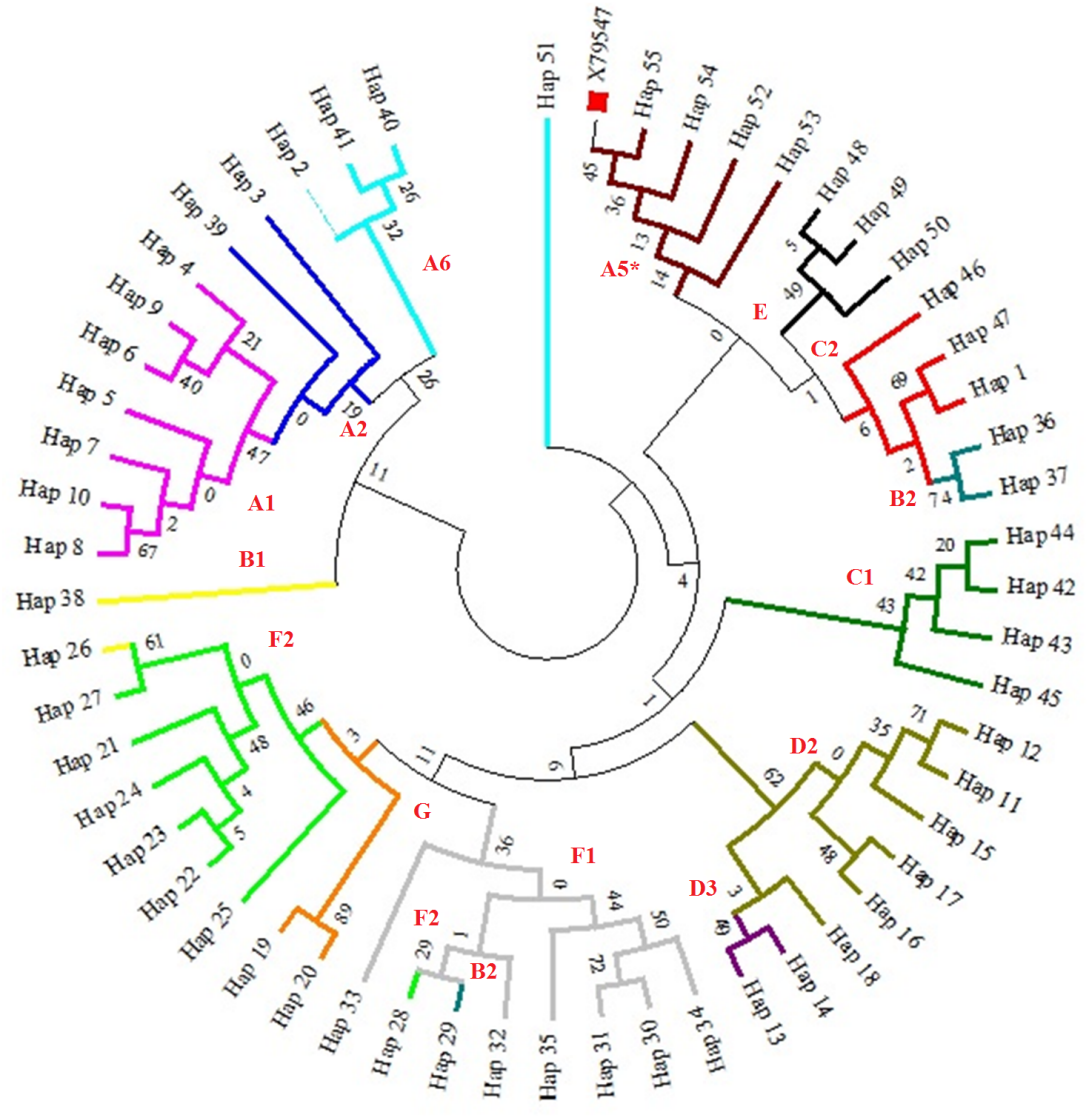
A maximum likelihood tree representing the phylogenetic relationships among 285 partial mtDNA D-loop horse sequences, including haplotypes of the Hungarian draft horses and the reference sequence X79547 (Xu & Arnason, 1994). The phylogenetic tree was based on the Tamura-Nei model of evolution with gamma distribution of rates and 1,000 bootstrap replicates (Tamura & Nei, 1993). Different colors represent major haplogroups according to the following: A1 (light purple), A2 (dark blue), A5 (brown), A6 (light blue), B1 (yellow), B2 (turquoise), C1 (dark green), C2 (red), D2 (mustard yellow), D3 (dark purple), E (black), F1 (grey), F2 (light green), G (orange). The red square represents the reference sequence.

We classified the resulting haplotypes into haplogoups, (Fig. 1) as previously defined by (Jansen et al., 2002). Detect fourteen of the seventeen haplogroups previously described in horses. Haplogroup D2 proved to be very common: it including 41 individuals and six haplotypes (14.74%). However, it is important to note that two haplotypes with four mares showed two variable positions within the G haplogroup, which is fairly rare. During the process of comparing Genbank sequences to our sequences no new mutations were found. The number of nucleotide differences and Kimura two-parameter distances were calculated among fifty-five mtDNA haplotypes. The Kimura two-parameter distances among haplotypes ranged from 0.005 to 0.063.

### Genetic differentiation of different horse breeds based on mtDNA D-loop sequence

Our purpose was to explore the mitochondrial genetic relationships between European horse breeds based on the sequences determined in our 285 individuals and a total of 419 sequences downloaded from the Genbank database. These added up to 704 different sequences, representing 52 different breeds, including our 285 individual Hungarian cold-blooded/Hungarian draft horses. The breeds represent a wide geographic area as well as different horse types. We tried to select cold-blooded varieties, which play an important role in developing the breeds like Breton, Noriker, Belgian cold-blooded or Percheron, and also included other common horse breeds like Akhal Teke, and Shetland Pony. Hungarian draft is a horse breed with a recent mixed ancestry. It was developed in the 1920s by crossing local mares with draught horses imported from France and Belgium. The foundation stock of this breed was initially the native Hungarian mares which were made to breed with other various breeds like the Noriker, Percheron, Ardennes and also with the native Hungarian stallions.

**Figure 2:**
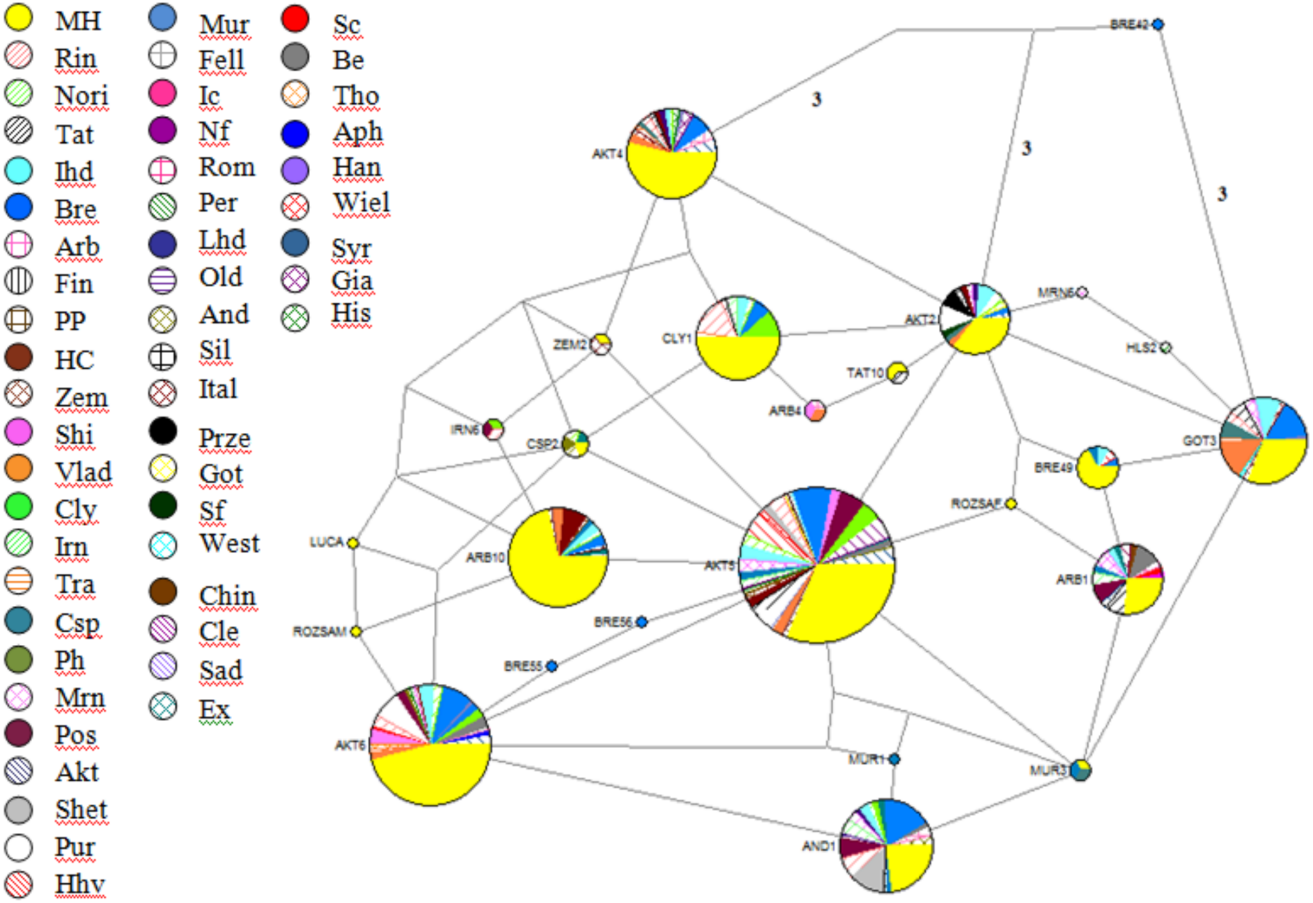
Reduced-Median-Joining network depicting relationships between horse haplotypes. Included are sequences of the 285 Hungarian draft individuals analysed in this study, plus sequences of European breeds available in the Genbank database. Sectors are proportional to the frequency of each haplotype. Branch lengths are not proportional to the mutational steps, the unmarked places means one, while others marked near branches. In the following order, acronyms, breed names, and sample sizes-indicated by ‘()’-were as follows: MH, Hungarian draft, our samples (number of samples: 285); Rin, Rhineland Heavy draft (25); Nori, Noriker (10); Tat, Turkoman Akhal Teke (19); Ital, Italian heavy draught (27); Bre, Breton (58); Arb, Arabian (10); Fin, Finn horse (2); PP, Polish primitiv (3); HC, Hucul (10); Zem, Zemaitukai heavy type (7); Shi, Shire (10); Vlad, Vladimir draught horse (21); Cly, Clydesdale (17); Irn, Iranian (14); Tra, Trakehner (4); Csp, Caspian Pony (5); Ph, Polish heavy (3); Mrn, Maremmano (15); Pos, Posavina (20); Akt, Akhal teke (16); Shet, Shetland pony (12); Pur, Pura Raza Espanola (17); Hhv, Croatian heavy draft (11); Mur, Murinsulaner (8); Fell, Fell (2); Ic, Icelandic Horse (2); Nf, Norwegian Fjord (2); Rom, Romanian draft horse (1); Per, Percheron (3); Lhd, Lithuanian heavy drought (3); Old, Oldenburg (1); And, Andalusian (2); Sil, Silesian (1); Ital, Italian (3); Sc, Scottish Highland (2); Be, Belgian (13); Tho, Thoroughbred horse (1); Aph, American Paint horse (1); Han, Hanovarian (3); Wiel, Wielkopolski (3); Syr, Syrian (5); Gia, Giara horse (2); His, Holstein (2); Prze, Przewalskii (3); Got, Gotland (3); Sf, Suffolk Punch (1); West, Westfalian (1); Chin, Chincoteague pony (1); Cle, Cleveland bay horse (11); Sad, Saddlebred (1); Ex, Exmoor pony (1).

These sequences were aligned and trimmed to obtain the above-mentioned 212-bp HVR I region. The Hungarian draft breed shared haplotypes with twenty other populations included in this analysis. It is noteworthy that Exmoor Pony, a breed known to have originated in the UK also shared haplotypes with two heavy horses, Italian Heavy and Rhineland Heavy, but not with the two other native UK cold-blooded breeds, Clydesdale and Shire. On the other hand, shared haplotypes among populations are indicators of common founder lineages or gene flow. A network of 25 oriental and European breeds was drawn up on the basis of mtDNA sequences (Jansen et al., 2002), which showed that from the total number of haplotypes (93) only nine included draft horses. The neighbor-joining tree with 419 Genbank sequences and 55 haplotypes from the present study could be divided into three clusters and contains haplogroups A-G. The consensus Neighbor-joining tree and the Median-joining network (Fig. 2) showed that individuals from different populations share identical haplotypes, indicating common ancestry and gene flow.

**Figure 3:**
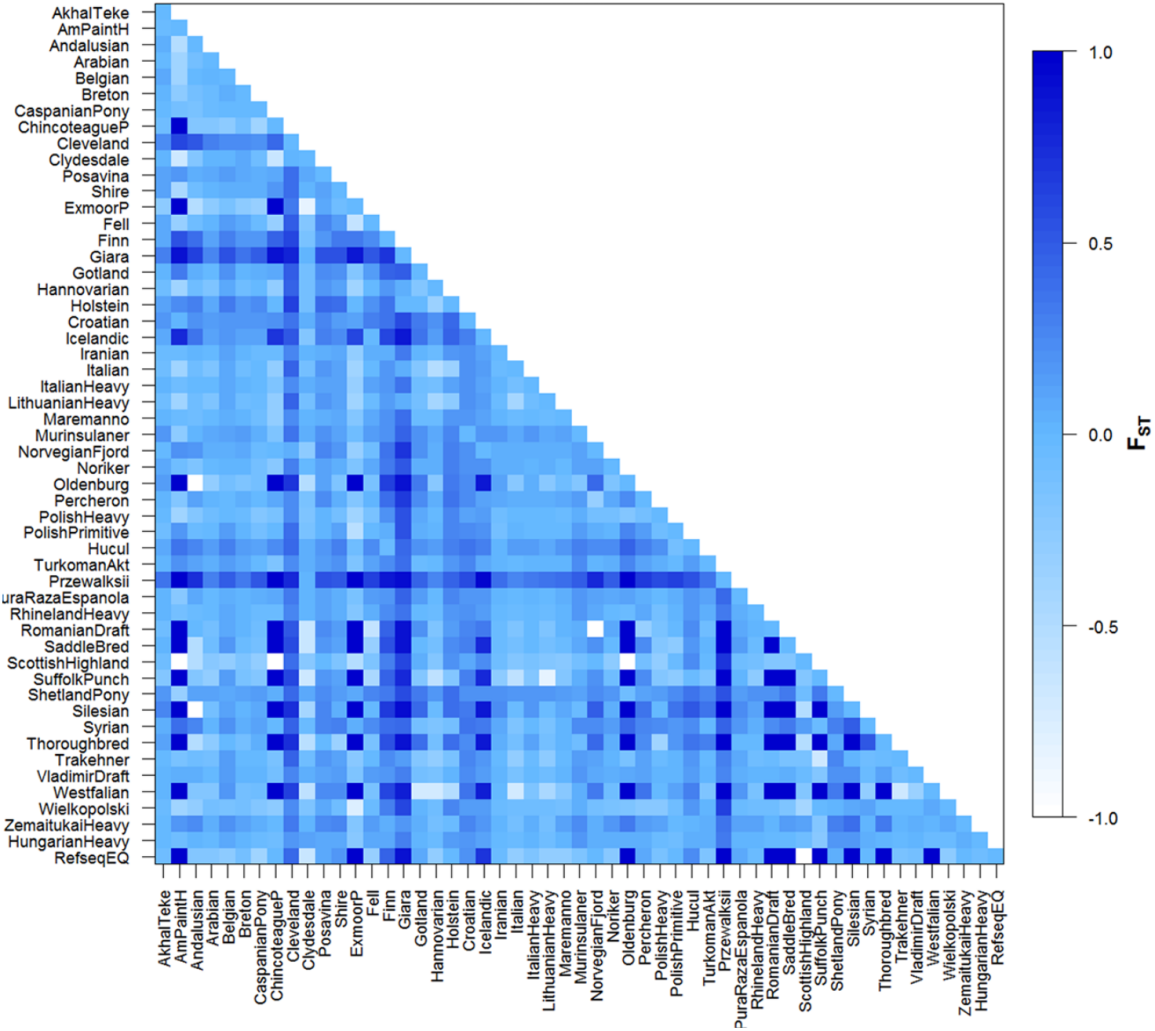
Graphic representation of pairwise *F*_ST_ values for all population comparisons. Significance level = 0.05. Graphic representation of different population relationships as described by *F*_ST_ computed between pairs of populations based on mtDNA datasets all of 212 base pairs. Shading reflects degree of divergence and corresponds to *F*_ST_ values indicated in legend (right).

Pairwise *F*_ST_ values are shown in Fig. 3. In some cases negative values were observed and these equate to zero *F*_ST_ values. Our *F*_ST_ values fall into a wide range, 0.00–1.00 (varying from white, and light to dark blue colors in Fig. 3). The *F*_ST_ comparison values obtained were significant in 492 pairwise calculations. Despite historic admixture with domestic horses and probably due to their low effective population size, Przewalskii could be differentiated from domesticated horses. Four breeds could be separated from only one other population on any level of significance, Italian-Cleveland Bay horse *F*_ST_ = 0.4461, Syrian-Polish Heavy Horse *F*_ST_ = 0.2217, Scottish Highland-Hucul *F*_ST_ = 0.2373, Wielkopolski-Finn horse *F*_ST_ = 0.3062, Croatian Heavy draft was significantly different from 34 other horse populations; on the other hand, this breed also has only a recent mixed ancestry, which in this case means relationship with 18 other breeds, mainly other cold-blooded horses. The Hungarian draft horse population was significantly differentiated from 12 other populations, namely: ShetlandPony, Przewalski, Hucul, Murinsulaner, Croatian Heavy draft, Giara horse, Belgian, Breton, Cleveland, Clydesdale, Posavina, Shire. Surprisingly, a significant difference was observed between Belgian horses and Hungarian draft, even though the import of cold-blooded Belgian Heavy horses started as early as before the First World War and led to the establishment of a cold-blooded flock in Hungary at that time (Becze, Lukáts & Zilahy, 1957).

## Discussion

In general, horse breeds are genetically close due to their somewhat recent domestication history. However, some breeds are highly differentiated due to strong bottlenecks, intensive selection and also isolation and genetic drift. There is no other farm animal species that exhibits a similar level of mitochondrial DNA variation (Cieslak et al., 2010). No genetic studies have been done on endangered Hungarian cold-blooded horses; therefore, the purpose of this work was to contribute to the characterization of the endangered Hungarian heavy draft horse populations in order to obtain useful information to implement conservation strategies for these genetic stocks. MtDNA analysis revealed multiple maternal origins, the absence of a population structure, and inbreeding. The reasons for the presence of such a large amount of genetic variation could have several explanations: multiple origins, large-scale introgression of local lineages into the domestic stock, or a large number of female founders (Cieslak et al., 2010).

The contents of A+T was the richest in the mtDNA D-loop region. It was in accordance with other studies, where A+T was 55.8%, whereas C+G was 44.2% (Zhang et al., 2012), and also matched the expected nucleotide composition of A>C>T>G with more A+T than G+C base pairs (Ji et al., 2008). Only three of the 36 detected polymorphisms showed insertions/deletions of single base pairs; there were 34 transitions and 1 transversion, which shows a shift towards transitions (Kim et al., 1999). The observed high haplotype and nucleotide diversity values proved to be greater than the values detected by Moridi et al. (2013) in Iranian horses, and less but quite similar to the diversity data of 0.975 and 0.977 reported in Pérez-Gutiérrez, De la Peña & Arana (2008) and Zhang et al. (2012), respectively. Direct comparisons with other studies have to be carefully considered, because different mtDNA regions were used in other reports. Fifty-five haplotypes were identified in our Hungarian cold-blooded samples. This number is quite similar to other findings reported for other horse breeds in Kavar et al. (1999) and Bowling, Del Valle & Bowling (2000). In the course of the haplogroup analysis of, we detected in our samples fourteen of those defined previously by Jansen et al. (2002). Four of our Hungarian draft horses belonged to haplogroup G, which is somewhat rare and may thus be a conservation target. Comparative research (McGahern et al., 2006) processing 962 sequences found just one archaic, 25 European, two Middle Eastern and two Far Eastern equines that contained variable positions which can be classified into haplogroup G. In a comprehensive study by Cardinali et al. (2016) they report that the second most common haplogroup was G with the highest values in the Giara. Based on the literature, haplogroups G appears to be more common in Asia and the Middle East in general. As we are lacking the general stud book in the case of Hungarian draft horse—although the Herd-book registration started in 1922—our goal is to determine whether the present stud book is appropriate. The first step is to classify mares of our existing flock into mare families. From the detected 55 haplotypes seven mare groups could be separated due to maximum likelihood and median joining network analysis. There were groups only with two haplorgoups, while the highest number in one mare family was seven. Phylogenetic network shows that Hungarian draftcould be separated from the following breeds (as there were no shared haplotypes with them): Exmoor Pony, Fell horse, Saddle bred, Croatian heavy draft, Murinsulaner, Przewalski and Silesian horse. The highest within-breed diversity was observed in breeds that are recently derived, as mentioned also by Petersen et al. (2013). We searched for shared haplotypes between the mentioned 52 different breeds and our samples. The Hungarian draft had shared haplotypes with 44 other breeds. Haplotypes from the same breed frequently clustered in separate groups that included breeds of completely different origins and breed types. The foundation stock of Hungarian draft was initially the native Hungarian mares which were crossed with other various breeds like the Noriker, Percheron, Ardennes and also with the native Hungarian stallions. Ten haplotypes were shared with each of Breton, Rhineland Heavy horse and Akhal Teke, and fourteen with Italian heavy horse. In the case of the cold-blooded horses examined, the haplotype named 27th was often shared. Our samples have three shared haplotypes with Hannoverian horses (three haplotypes), not unexpectedly, as this breed is known to be an outbred population, influenced by many different breeds from different regions (Aberle et al., 2007). Similar results were reported in mtDNA (Vilà et al., 2001). Although a significant difference was observed between Belgian horses and Hungarian draft, four haplotypes were shared between them, also four with Noriker and two with Percheron. Haplotype 3 was common between Percheron and Belgian, while haplotype 26 and 27 was found in Noriker and Belgian two. This indicates possible gene flow among those horse populations, or common ancestry, as it known that these breeds were used in crossing for better phenotypic appearance The genetic clustering analysis did not show any clear pattern of differentiation among all populations. *F*_ST_ analysis supports this weak pattern of differentiation showing high rates of mtDNA sharing between populations. This state is also confirmed by the observation that the median-joining network does not have a start-like structure, suggesting that a large number of founders could have produced the Hungarian cold-blooded breed. Secondly, a high percentage of the variability among pairwise *F*_ST_ values is explained by actual migrations demonstrating, further, strong relationship between population structure and observed gene flow. The haplotypes found in Hungarian cold-blooded horses were dispersed in the tree across several haplogroups. Mitochondrial lineage diversity changed over time by breeding or hybridization and introgression; as a result, a breed is not necessarily isolated from other populations (Cieslak et al., 2010). The Kimura two-parameter distances among haplotypes ranged from 0.005 to 0.063. This is as wide interval as observed in the case of other Hungarian horses like Hucul, where these distances ranged from 0.004 to 0.054 (Kusza et al., 2013). Also, these values indicate higher within-breed variation than the observed by Cothran, Juras & Macijauskiene (2005) in the Zemaitukai horse breed. Nucleotide sequence diversity ranged from 0.47% to 6.1%. Since maternal variability in domestic horse breeds is very high, they cannot be sharply distinguished from each other. Understanding the genetic diversity of equids and classifying their populations is essential for an appropriate conservation plan to be developed (Oakenfull, Lim & Ryder, 2000). Genetic diversity within and among breeds can also influence decisions affecting the breeds or species to be preserved (Thaon d’Arnoldi, Foulley & Ollivier, 1998). Greater mtDNA diversity was found in old Iberian breeds than in American breeds, although they have a recent mixed ancestry (Lira et al., 2010). It has been stated that the maintenance of small isolated groups is the choice management strategy to preserve variability (Toro & Caballero, 2005), which also needs scientific planning and a breeding plan. The results obtained with mitochondrial markers are consistent with a recent hybrid origin of the Hungarian breed. The high variability levels emphasize the importance of the conservation of this breed, as it can be an important reservoir of genetic biodiversity.

## Conclusion

Hungarian heavy draft counts 800 mares today, and only survives due to breeding programs; in this way, each haplotype frequency depends on the extent to which mares are involved in the breeding. Since breeders lack written documentation the present stud book contains unknown individuals in pedigree. However, we confirmed the multiple origins in the maternal lineage of domestic horse breeds reported by other researchers (Cieslak et al., 2010). We present high nucleotide and haplotype diversity values, no haplotypes clearly distinct from other populations, and no clear clustering on the median joining tree. Both heterozygosity and diversity levels were found to be high in this breed. Almost 40% of the Hungarian population were sampled, but it is unclear whether a further increase in sample size would add to the differentiating ability of the methodology, although mtDNA probably has no power to differentiate breeds (due to lack of genetic structure in horses). It is important that good management practices continue to ensure the survival of this breed of economic significance. Analyzing other markers to characterize Hungarian horses, e.g., autosomal variation, could contribute to a more comprehensive study of Hungarian heavy draft horses. The results presented here could be regarded as a genetic portrait of the maternal lineages of Hungarian cold-blooded horse population.

##  Supplemental Information

10.7717/peerj.4198/supp-1 Table S1 Polymorphic sites in the control region of the Hungarian draft horse population sequenced Nucleotide positions 15,531–15,752 as compared to GenBank reference sequence X79547 (Xu & Arnason, 1994).

10.7717/peerj.4198/supp-2 Table S2 Sample size (n), total number of haplotypes and polymorphic sites, mean number of pairwise differences between haplotypes (MNPD), number of transitions, transversions and indels, nucleotide diversity (*π* ± SD), haplotype diversity (Hd) observed in each of 52 horse populations

10.7717/peerj.4198/supp-3 Table S3 Individuals belong to haplotypes

10.7717/peerj.4198/supp-4 Data S1 Raw data for 285 Hungarian draft sequences

## Additional Information and Declarations

### DNA Deposition

The following information was supplied regarding the deposition of DNA sequences: GenBank accession numbers of D-loop haplotypes: KY512807 to KY513091.

### Data Availability

The following information was supplied regarding data availability: The raw data has been provided as a Data S1.
