# Supplementary material for: Genetic analysis of the Hungarian draft horse population using partial mitochondrial DNA D-loop sequencing
Source: PeerJ. 2018 Jan 31;6:e4198. doi: 10.7717/peerj.4198 (PMC5797449; doi:10.7717/peerj.4198)
Supplement: Table S1 — Nucleotide positions 15,531–15,752 as compared to GenBank reference sequence X79547 (Xu & Arnason, 1994). [file peerj-06-4198-s001.docx]

**Polymorphic sites in the control region of the Hungarian draft horse population sequenced** Nucleotide positions 15531–15752 as compared to GenBank reference sequence X79547 (Xu & Arnason, 1994).

 Sequence identity is indicated by ‘.’, gaps by ‘-‘. ‘*’ mean reference sequence.
