# Supplementary material for: Genetic analysis of the Hungarian draft horse population using partial mitochondrial DNA D-loop sequencing
Source: PeerJ. 2018 Jan 31;6:e4198. doi: 10.7717/peerj.4198 (PMC5797449; doi:10.7717/peerj.4198)
Supplement: Table S2 [file peerj-06-4198-s002.docx]

**Sample size (n), total number of haplotypes and polymorphic sites,** **mean number of pairwise differences between haplotypes (MNPD), number of transitions, transversions and indels, nucleotide diversity (*π* ± SD), haplotype diversity (Hd) observed in each of 52 horse populations.**

| Population | n | Polymorphic sites | Haplotypes | Transitions | Transversions | Indels | Nucleotide diversity | Sd | Mean number of pairwise differences | Sd | Haplotype diversity |
| --- | --- | --- | --- | --- | --- | --- | --- | --- | --- | --- | --- |
| **Akhal Teke** | 16 | 19 | 14 | 19 |  |  | 0.0321 | 0.0182 | 5.6431 | 2.8569 | 0.9833 |
| **Arabian** | 10 | 20 | 9 | 20 |  |  | 0.0419 | 0.0242 | 7.3749 | 3.7701 | 0.9778 |
| **Belgian** | 13 | 17 | 8 | 14 |  | 3 | 0.0264 | 0.0155 | 4.7185 | 2.4690 | 0.8974 |
| **Breton** | 58 | 35 | 29 | 25 | 12 |  | 0.0354 | 0.0189 | 6.2351 | 3.0039 | 0.9595 |
| **Caspanian Pony** | 5 | 13 | 5 | 13 |  |  | 0.0329 | 0.0222 | 5.7955 | 3.3364 | 1 |
| **Cleveland bay horse** | 11 | 6 | 3 | 6 |  |  | 0.0110 | 0.0076 | 1.9352 | 1.1877 | 0.4727 |
| **Clydesdale** | 17 | 169 | 9 | 42 | 127 | 11 | 0.0111 | 0.0758 |  |  | 0.9044 |
| **Croatian heavy draft** | 11 | 14 | 9 | 11 | 2 | 1 | 0.0253 | 0.0152 | 4.4733 | 2.3861 | 0.9636 |
| **Gotland** | 3 | 6 | 3 | 6 |  |  | 0.0233 | 0.0197 | 4.0979 | 2.7845 | 1 |
| **Hanovarian** | 3 | 11 | 3 | 11 |  |  | 0.0436 | 0.0349 | 7.6676 | 4.9277 | 1 |
| **Hucul** | 10 | 10 | 4 | 10 |  |  | 0.0244 | 0.0149 | 4.2817 | 2.3158 | 0.7111 |
| **Hungarian draft** | 285 | 36 | 55 | 34 | 1 | 3 | 0.0342 | 0.0181 | 6.0120 | 2.8738 | 0.9544 |
| **Iranian** | 14 | 22 | 14 | 22 |  |  | 0.0335 | 0.0191 | 5.9019 | 2.9984 | 1 |
| **Italian** | 3 | 11 | 3 | 11 |  |  | 0.0437 | 0.0350 | 7.6856 | 4.9386 | 1 |
| **Italian heavy draft** | 27 | 26 | 22 | 26 |  |  | 0.0320 | 0.0176 | 5.6265 | 2.7859 | 0.9829 |
| **Lithuanian Heavy** | 3 | 11 | 3 | 11 |  |  | 0.0438 | 0.0351 | 7.7129 | 4.9549 | 1 |
| **Maremanno** | 15 | 22 | 12 | 22 |  |  | 0.0328 | 0.0187 | 5.7773 | 2.9288 | 0.9619 |
| **Murinsulaner** | 8 | 17 | 8 | 13 | 3 | 1 | 0.0404 | 0.0242 | 7.1515 | 3.7539 | 1 |
| **Noriker** | 10 | 15 | 6 | 15 |  |  | 0.0297 | 0.0177 | 5.2192 | 2.7576 | 0.8889 |
| **Norvegian Fjord** | 2 | 4 | 2 | 4 |  |  | 0.0233 | 0.0260 | 4.0938 | 3.2289 | 1 |
| **Percheron** | 3 | 6 | 3 | 6 |  |  | 0.0234 | 0.0198 | 4.1104 | 2.7920 | 1 |
| **Polish Heavy** | 3 | 9 | 3 | 9 |  |  | 0.0353 | 0.0288 | 6.2187 | 4.0591 | 1 |
| **Polish Primitiv** | 3 | 7 | 3 | 7 |  |  | 0.0273 | 0.0228 | 4.8216 | 3.2201 | 1 |
| **Posavina** | 20 | 18 | 12 | 18 |  |  | 0.0251 | 0.0145 | 4.4305 | 2.2806 | 0.9368 |
| **Pura Raza Espanola** | 17 | 15 | 14 | 15 |  |  | 0.0274 | 0.0158 | 4.8333 | 2.4811 | 0.9779 |
| **Rhineland Heavy** | 25 | 22 | 16 | 22 |  |  | 0.0344 | 0.0189 | 6.0634 | 2.9873 | 0.9267 |
| **Scottish Highland** | 2 | 6 | 2 | 6 |  |  | 0.0353 | 0.0380 | 6.2143 | 4.7345 | 1 |
| **Shetland Pony** | 12 | 13 | 5 | 12 | 1 |  | 0.0279 | 0.0165 | 4.9216 | 2.5775 | 0.6667 |
| **Shire** | 10 | 15 | 8 | 15 |  |  | 0.0287 | 0.0173 | 5.0649 | 2.6850 | 0.9556 |
| **Syrian** | 5 | 8 | 5 | 8 |  |  | 0.0210 | 0.0149 | 3.7033 | 2.2437 | 1 |
| **Trakehner** | 4 | 14 | 4 | 14 |  |  | 0.0425 | 0.0301 | 7.4967 | 4.4393 | 1 |
| **Turkoman Akhal Teke** | 19 | 18 | 13 | 18 |  |  | 0.0280 | 0.0159 | 4.9316 | 2.5117 | 0.9532 |
| **Vladimir Draft** | 21 | 24 | 14 | 24 |  |  | 0.0339 | 0.0188 | 5.9746 | 2.9675 | 0.9476 |
| **Wielkopolski** | 3 | 7 | 3 | 7 |  |  | 0.0273 | 0.0227 | 4.8048 | 3.2100 | 1 |
| **Zemaitukai Heavy** | 7 | 14 | 6 | 14 |  |  | 0.0319 | 0.0199 | 5.6193 | 3.0684 | 0.9524 |
