# Supplementary material for: Genetic analysis of the Hungarian draft horse population using partial mitochondrial DNA D-loop sequencing
Source: PeerJ. 2018 Jan 31;6:e4198. doi: 10.7717/peerj.4198 (PMC5797449; doi:10.7717/peerj.4198)
Supplement: Table S3 [file peerj-06-4198-s003.docx]

| **Haplotypes** | **Number of mares** | **Individuals** |
| --- | --- | --- |
| 1 | 1 | Farsan |
| 2 | 1 | Julcsi |
| 3 | 4 | Orsi, Harmat, Jumi, Galusk |
| 4 | 1 | Man177 |
| 5 | 1 | Kato |
| 6 | 2 | BabN22, ManN9 |
| 7 | 1 | Fiona |
| 8 | 4 | Emma, Lena, Sar71, Lin181 |
| 9 | 19 | Kati, Vihar, Barbi, Csn188, Luj204, Molli, Rez253, Regina, Bar40, Pan53, Röpke, Lola, Kat141, Büszke, Timea, TimN36, HolN29, B_bajo, Otilia |
| 10 | 3 | Matka, Reb92, Stella |
| 11 | 1 | Rita |
| 12 | 12 | Bog119, Bet126, Cseng_, Cs_pi, Ste180, Sac187, Laura, Man205, Helga, Csa130, Donna, Hollo |
| 13 | 1 | Csabit |
| 14 | 24 | Cumis, Sar109, Lin124, Szilvi, Ejnye, Egysze, Hazug, szike, Fec189, Bibor, Sze235, Moni, Fani, Szonja, Uborka, Uritök, Uszo, Debora, Fatany, Sari, Holdfe, Villam, Bolygo, Vera |
| 15 | 3 | Csinta, Mok236, Pici |
| 16 | 1 | Eger1 |
| 17 | 8 | Enikő, Etna, Emese, Eszter, Emma55, Edit, Eszti, Ercsi |
| 18 | 16 | Betti, Baj145, Szomor, Rit198, Rifke, Stefi, Lepke, Mara, Utopia, BolN41, Barsony, Remény, Danka, Geszte, Hajni, Happy |
| 19 | 1 | Rig259 |
| 20 | 3 | Kitti, Dama, Kat24 |
| 21 | 1 | Szepla |
| 22 | 5 | Csn221, Kismac, Fanta, Golya, N42 |
| 23 | 1 | Honvag |
| 24 | 2 | Bősz89, Kat63 |
| 25 | 1 | Kat152 |
| 26 | 2 | Rozi, Kata |
| 27 | 1 | Karmen |
| 28 | 1 | RozsaM |
| 29 | 1 | Luca |
| 30 | 2 | Inci, Habane |
| 31 | 1 | Olga |
| 32 | 1 | Bet13 |
| 33 | 5 | Bea, Cs_rf_, Csarda, Bibork, Dori |
| 34 | 30 | Dicső, DudN47, Ultima, Reb251, Dud36, Igeret, Sar60, Elza, Ancsa, Em_ke, Dedos, Eminen, Ibolya, Eger, Rayi, Cilike, Degesz, Doxa, Diadem, Dama52, Deszep, Csal64, Szandr, Csg79, Cini, Fanni, FanN40, Hetyke, Paulin, Panni |
| 35 | 6 | Darido, Monda, Rit69, Mon73, Gondos, Babilo |
| 36 | 22 | Mese, Szeles, Mokany, Rona, Füstös, Csa232, Boroka, Rem67, Rigo, Ari, Saci, Hilda, Hanga, Ron239, Aniko, Lizi, Törpe, Tanya, Izaura, Gierl, Bodza, Felleg |
| 37 | 1 | Bogi |
| 38 | 2 | Urilan, Unikum |
| 39 | 3 | Lujza, Szell_, Lenke |
| 40 | 5 | Zsembe, Dolli, Zsofi, Bet68, Tündi |
| 41 | 9 | Ste48, Vilma, Hamvas, Fatyol, Tüzes, Veroni, Cuki, Magdi, BabaN3 |
| 42 | 2 | Panka, Pomade |
| 43 | 1 | Giz138 |
| 44 | 21 | Mirza, Len106, Vil140, Csn142, Kifli, Lili, Marika, Mozdon, Ludver, Len27, Lin6, Man7, Lin86, Fecske, Hovira, Leanyk, Gy_ngy, Gracia, Merced, Mania, Mimoza |
| 45 | 6 | Kincs, Kabala, Utas, Arvacs, Abrand, Csg270 |
| 46 | 2 | Robert, Virag |
| 47 | 15 | Edina, Bajos, Gizi, Holl186, Rum, Baba, Ella, Roz257, Dorci, Eszmer, Csi110, Juci, Rozsa, Janka, Reka |
| 48 | 1 | Sors |
| 49 | 5 | Csg195, Cudar, Csilla, Bebi, Andi |
| 50 | 2 | Csinos, Manci |
| 51 | 2 | Vercse, Csn242 |
| 52 | 1 | Böbe |
| 53 | 1 | Rozsaf |
| 54 | 8 | Merci, Vir45, Ursula, Ultra, Utazo, Linda, UboN14, Szelit |
| 55 | 9 | Almos, Ludmil, Izabel, T_n250, Laur32, Agota, Almodo, Nora, Kacer |
